# Welcome to the era of vague news: a study of the demands of statistical and mathematical products in the COVID-19 pandemic media

**DOI:** 10.1007/s10649-022-10151-7

**Published:** 2022-04-25

**Authors:** Iddo Gal, Vince Geiger

**Affiliations:** 1 Department of Human Services, University of Haifa, Health Sciences Building, Room 207, 199 Aba Khoushy Ave., 3498838 Haifa, Israel; 2 Institute for Learning Sciences and Teacher Education, Australian Catholic University, Brisbane, Australia

**Keywords:** Adult numeracy, Statistical literacy, Mathematics education, Critical interpretation, Mathematics in the media, Evidence

## Abstract

In this article, we report on a typology of the demands of statistical and mathematical products (StaMPs) embedded in media items related to the COVID-19 (coronavirus) pandemic. The typology emerged from a content analysis of a large purposive sample of diverse media items selected from digital news sources based in four countries. The findings encompass nine categories of StaMPs: (1) descriptive quantitative information, (2) models, predictions, causality and risk, (3) representations and displays, (4) data quality and strength of evidence, (5) demographics and comparative thinking, (6) heterogeneity and contextual factors, (7) literacy and language demands, (8) multiple information sources, and (9) critical demands. We illustrate these categories via selected media items, substantiate them through relevant research literature, and point to categories that encompass new or enhanced types of demands. Our findings offer insights into the rich set of capabilities that citizens (including both young people and adults) must possess in order to engage these mass media demands, critically analyze statistical and mathematical information in the media, evaluate the meaning and credibility of news reports, understand public policies, and make evidenced-informed judgments. Our conclusions point to the need to revise current curricular frameworks and conceptual models (e.g., regarding statistical and probability literacy, adult numeracy), to better incorporate notions such as blended knowledge, vagueness, risk, strength of evidence, and criticality. Furthermore, more attention is needed to the literacy and language demands of media items involving statistical and mathematical information. Implications for further research and educational practice are discussed.

## Introduction

The mass media is the primary vehicle through which most citizens are informed of current affairs on key social and economic matters (Miller & Krosnick, 2000). Consequently, the critical interpretation of media reports has been a focus of research within mathematical literacy, adult numeracy, and statistical literacy (Jablonka, 2003; 2015). The rapid progress of the COVID-19 pandemic has impacted on many facets of life, prompting governments to both monitor and make predictions about the crisis in order to formulate and enact evidence-based public policy aimed at protecting their citizens. This has required the analysis of vast amounts of quantitative data and information. As a result, citizens have been exposed to a wide range of pandemic-related media items published by various actors in the public and private sphere.

In this article, we use *media items* as an umbrella term for diverse types of publically accessible news. Media items appear in print-based newspapers and magazines, digital media (e.g., news websites, expert television interviews, announcements by public officials), radio broadcasts, blogs, podcasts, and postings on social networks by official organizations and other actors. Such media items include *Statistical and Mathematical Products* (StaMPs) that place interpretive and evaluative demands on a reader or viewer. StaMPs encompass all the diverse statistical and mathematical entities or ideas that appear in media items within the public sphere (in our case, COVID-19 pandemic issues). These include, for example, rates, the results of mathematical computations or modelling, and descriptions of trends and projections. StaMPs may be communicated via any combination of texts (written or spoken), numbers, and/or visual representations. StaMPs can be used to: inform the public; present or support opinions; justify the predictions and recommendations by officials, experts, or commentators; and validate government policy decisions. Thus, any media item may combine both StaMPs and other elements that do not touch on mathematical or statistical topics and ideas. In developing the notion of StaMPs, we draw on aligned but distinct scholarly perspectives regarding the differences and commonalities between mathematics and statistics (e.g., Cobb & Moore, 1997).

Through this article, we outline and substantiate the construct of StaMPs, providing new insights into the following perennial research and policy question (European Commission, 2018; Madison & Steen, 2003):

What mathematical and statistical capabilities must adults and young people possess in order to comprehend and critically evaluate the StaMPs appearing within media items?

Despite the importance of such capabilities, we argue that there has been limited systematic research on the actual demands of media items with which adults need to engage. Our goal is to begin addressing this knowledge gap since identifying the characteristics of StaMPs and their associated demands, is essential for revising current conceptual frameworks for vital adult capabilities or basic skills, making educational decisions, and designing effective curricula. Furthermore, since StaMPs have been used in the media to document and predict the impact of the pandemic on many facets of life across the globe, our typology may be relevant to the evaluation of other current or future national and international challenges and crises (ProCivicStat Partners, 2018).

The article is organized into five sections. Following the introduction, we review relevant literature related to responsible citizenship and the use of StaMPs in the news media (Section 2). Our approach to choosing and analyzing a large, multinational sample of media items is outlined in Section 3. We then report on the findings of our analysis in Section 4. In Section 5, four main contributions of the study are discussed, alongside implications for theory and practice.

## Responsible citizenship and StaMPs in the news media

In this section, we review research literature related to the role of StaMPs in constructing and supporting claims made in the news media, and to the associated capabilities required for informed, critical, and responsible citizenship and for responding to changing government guidelines aimed at reducing the spread and impact of the pandemic.

Although the role of mathematics in the news media has been of interest for close to 100 years (e.g., Bertotti, 1941; Sueltz, 1931), limited systematic research has been conducted on the mathematical and statistical demands of mainstream media. Previous empirical studies have usually been confined to the (pre-digital) print media (e.g., Joram et al., 1995) and focused on selective topics such as the use of graphs or particular numerical content in the news (e.g., Himmelstein, 2014), or on health education (Golbeck et al., 2005) and financial literacy (Lusardi & Mitchell, 2017).

At the same time, the capabilities adults need to act as “smart consumers” of the news media have been the subject of significant theory development, including conceptual frameworks that describe key competencies, such as adult numeracy and statistical literacy—these encompass multiple knowledge bases and dispositions (Gal, 2002; Geiger et al., 2015; PIAAC Numeracy Expert Group, 2009; Tout et al., 2021). In fields such as journalism and political science, interest in “numbers in the media” has generated suspicion that numbers are often used only as “rhetorical devices” that appeal to readers’ emotions, prejudices, and fears (e.g., Roeh & Feldman, 1984). Such devices have been identified as pivotal within media constructions of social problems (Himmelstein, 2014) and the exercise of political power (Rosa et al., 2016; Rose, 1991).

There has also been an increasing emphasis in the literature on the need for citizens to develop *critical* capabilities. Calls have been made for educational approaches that can promote an understanding of how mathematics and statistics are used to serve social power structures, or manipulate and shape public opinion (e.g., Frankenstein, 1989; Geiger, et al., 2020; Weiland, 2017). For example, the focus of a recent essay by Skovsmose (2021) describes three different relationships between mathematics and crises (such as the current pandemic): (1) modelling or picturing of crises as a way of representing reality; (2) construing a crisis in that mathematics is integrated into the development of a phenomenon; and (3) formatting a crisis in a way where mathematics is used to shape the reading and interpretation of a crisis. The third relationship is particularly relevant to the ways in which StaMPs are used by the media in shaping a readers’ interpretation of developments related to the COVID-19 pandemic.

The COVID-19 crisis has seen StaMPs used in the media (both print and digital) not just for descriptive or predictive purposes, but also as evidence to justify unprecedented interventions by governments into citizens’ daily lives, such as the restriction of travel and work options, social distancing, and the use of digital data for surveillance (Zuboff, 2019). StaMPs have also been used to explain the impact of the pandemic on the economy (e.g., layoffs, business closures) and to justify why public resources must be diverted to new causes. Citizens’ compliance with government directions has saved lives but has also had detrimental financial, health, and personal consequences. If citizens from all walks of life, including from vulnerable groups (Gal et al., 2020), are to make informed decisions and act responsibly within a pandemic context, they must be capable of critically evaluating the meanings and credibility of StaMPs within media items (cf. Glik, 2007; Steen, 1999). They should also be able and willing to engage with opinions of experts, ask relevant questions, evaluate the quality of given evidence, draw conclusions, and make decisions (Fischer, 2000; Kollosche & Meyerhöfer, 2021).

The complex social, economic, and political ecology associated with the COVID-19 pandemic has caused a resurgence of interest in the capabilities required to comprehend and (critically) interpret quantitative information that is accessible to the public. Since mid-2020, over fifty new relevant articles have been published in peer-reviewed journals on mathematics and statistics education. Through the lens of the research question posed earlier, such articles can be divided into three groups. The first group is a large group that presents scholarly reflections about the capabilities required to comprehend selected mathematical or statistical ideas, such as modelling, mortality rates, and exponential growth (e.g., Best, 2020; Borba, 2021; Kollosche & Meyerhöfer, 2021; Reiss, 2020; Ridgway, 2021). The second group includes articles about studies of how target groups, such as students, adults, or teachers, understand selected mathematical or statistical topics in the media (e.g., Heyd-Metzuyanim et al., 2021).

The third group, which is particularly relevant to the current study, includes reports on *systematic* analyses of media items. An extensive search has so far identified only three such articles: Kwon et al. (2021) analyzed the demands of graphs (only) in seven South Korean newspapers; Aguilar and Castaneda (2021) utilized a sample of 25 video-based daily reports from the Mexican Ministry of Health; and Jablonka and Bergsten (2021) canvassed 40 media items from a single news website in Germany. While innovative and insightful, these studies have either focused on a single mathematical topic or used a single media source, often employing a pre-existing conceptual framework to guide a content analysis of data. In this study, we present a systematic analysis that led to a new typology based on categories of StaMPs within pandemic-related media items.

## Approach

In order to develop a comprehensive empirically based typology of the characteristics of StaMPs in pandemic-related media items, we have adopted a rigorous exploratory approach to data collection. Our approach extends, or differs from, previous research on the demands of COVID-19 media in the following ways: (1) content analysis was conducted on a larger sample of 300 media items, published on the websites of four leading news outlets from different countries (see Table 1), with heterogeneous demographic, geographical, and economic characteristics and different patterns of how the pandemic evolved in each country; (2) multiple media outlets were purposefully selected that represent diverse political orientations (left, right, and centrist) *and* appeal to heterogeneous audiences; and (3) the salience of chosen media items for readers was purposefully enhanced by selecting about half of the items from lead or section-lead articles (i.e., appeared as the headline or a highlighted article on the outlet’s website for a specific day), others from key sections related to news, health, COVID-19, and business.

**Table 1:** Characteristics of chosen news outlets

Source	Outlet characteristics
The Australian Broadcasting Corporation (*ABC*)	A public national broadcaster with international reach. Viewed as a highly credible source about current affairs. Seen by some to present a left-of-center political perspective
Yedioth Ahronoth (*YNET*, Israel)	The largest circulation print-based newspaper in Israel (Hebrew-based), considered centrist and investigative. Provides coverage of a wide range of social and economic topics
*CNN* news network (International Edition), USA	A US-based media outlet reporting on international events. Considered to be both credible and reliable, with a left-of-center political perspective
*The Sun*, UK	The largest circulation newspaper in the UK. Seen as a populist media outlet with a right-of-center political perspective

All media items used in the current analysis appeared within a 4-month timeframe of 15 March to 15 July 2020, covering the general period in which most countries faced the first wave of the pandemic, similar to earlier studies, and a period during which media outlets in different countries published thousands of articles pertaining to COVID-19 (see Jablonka & Bergsten, 2021; Kwon et al., 2021). We chose a starting point of 15 March 2020, which was one month after the first reported death from COVID-19 in Europe (15 February, in France), to allow for sufficient data about infections and deaths to accumulate and for mathematical and statistical projections and media discourse to evolve in different countries and globally.

Consistent with the explorative nature of our research question, our approach to the content analysis of selected media items was reductive, by way of inductive category formation (Mayring, 2014). Krippendorff (2004) suggests that this method is appropriate when different authors write texts about a common theme—as is the case with articles about pandemic-related issues. The process of induction was initiated by a first pass over a subsample of media items, consistent with an open coding technique drawn from grounded theory (Strauss & Corbin, 1998). This pass was aimed at identifying tentative categories of mathematical and statistical entities or ideas within each media item. The approach was informed by, but not limited to, ideas drawn from conceptual frameworks that describe foundational elements of statistical literacy, civic statistics, and adult numeracy (e.g., Gal, 2002; PIAAC Numeracy Expert Group, 2009; ProCivicStat Partners, 2018; Tout et al., 2021). Emergent broad categories of StaMPs were identified and defined, including several ideas not included in such prior frameworks. Then, additional items in the sample were examined, using a process involving constant comparison and ongoing discussion between the researchers, in order to refine the emerging categories. Consistent with Mayring’s (2015) approach, this process was re-applied, leading to further refinement (reducing redundancy, overlapping or lack of clarity), until all categories and their definitions stabilized.

## Findings: Mathematics and statistics demands in pandemic-related media items

The analysis of our large sample led to the identification of a typology of nine categories of StaMPs that appeared in media items about the pandemic. These categories cover the following:

1. Descriptive quantitative information
2. Models, predictions, causality and risk
3. Representations and displays
4. Data quality and strength of evidence
5. Demographics and comparative thinking
6. Heterogeneity & contextual factors
7. Literacy and language demands
8. Multiple information sources
9. Critical demands

We see these categories as discrete but often interrelated, since a single media item may encompass multiple StaMPs. For example, the results of a model (category 2) may be communicated in media items via spoken texts (category 7) or visual representations (category 3). Category 9, critical demands of StaMPs, however, is broader and can be relevant to or applied to any of the other eight categories when interrogating a media item.

In the subsections that follow, these nine categories of StaMPs are described, illustrated via examples from media items, and substantiated through reference to relevant research literature. Given space constraints, categories 5–8 are sketched in broad strokes only, although each is rich and deserves further elaboration. (Note: Media items are referenced with author names and year of publication, and preceded by a sequential number, e.g., ^Item#1^. Citations for all media items are grouped in the closing “Media items” section).

### Category 1: Descriptive quantitative information

The first category of StaMPs that emerged from the content analysis involves *descriptive quantitative information*. This category encompasses diverse types of seemingly basic figures and statistics regarding four areas sketched in Table 2: the status and/or progression of the pandemic; status of health systems; impact of the pandemic on economic and employment issues; and impact of the pandemic on other societal issues. Taken together, these serve to inform the public of “what is going on” with the pandemic.

**Table 2:** Four areas of descriptive quantitative information regarding the pandemic

	Area	Examples
A	Status and/or progression of the pandemic	Figures related to deaths per day, people in self-quarantine, testing, levels of infection, those hospitalized or in intensive care. Statistics about infection rates—overall or for subgroups, such as comparing older (e.g., over 70) and younger cases, or geographical locations
B	Status of health systems	Figures about available beds, infected medical personnel or in quarantine, equipment availability (e.g., ventilators, face masks)
C	Impact of the pandemic on economic and employment issues	Discussion of figures and trends related to business losses and closures, workers laid off, product shortages, supply-and-demand concerns, and government interventions (e.g., tax cuts, financial aid for companies or individuals)
D	Impact of the pandemic on other societal issues	Figures related to students learning from home, vulnerable people living in nursing homes, welfare issues (e.g., rise in domestic abuse cases, calls to health lines) and changes in environmental indicators (e.g., lower pollution levels due to less traffic)

Our analysis also identified three nuances on the ways media items are using descriptive quantitative information. First, and not surprisingly given prior literature (Whitacre et al., 2020), descriptive quantitative information often involved mathematical entities typically associated with arithmetic or number sense, for example, counts, totals, percentages, proportions and rates. As the pandemic progressed, however, descriptive figures were reported in a *cumulative* and *comparative* fashion, since public discourse about the pandemic’s status, or national strategies for slowing the progress of the pandemic, involved comparing relative rates of infection or deaths across time, locations and demographic groups.

Second, key *statistical indicators* (ProCivicStat Partners, 2018) were discussed in media sources from all four outlets in a descriptive fashion, including R rate (reproduction or replication rate), growth rate and positivity rate (We note that statistical indicators by themselves are models of phenomena; Gal & Ograjenšek, 2017). While there are attempts to explain the meaning of these indicators in some reports, as illustrated in *The Sun's* “Corona explained” section (^Item#4^Jones & Steed, 2020) in Table 3, often readers are left to interpret them themselves.

**Table 3:** Explanation about the R rate, *The Sun*

What is the R rate and how is it estimated?
“R0…refers to the average number of people that one infected person can expect to pass the Coronavirus on to. Scientists use it to predict how far and how fast a disease will spread—and the number can inform policy decisions about how to contain an outbreak
For example, if a virus has an R0 of three, it means that each sick person will pass the disease on to three more people—if no containment measures are introduced
If the R rate of a disease is one or above, it will spread exponentially, infecting more and more people. Therefore, being below R1 is key because it means the virus will likely peter out
Scientists work backwards to estimate the R rate, as we can’t know the exact moment people are infected. A range of data is used, such as ICU admissions, hospital admissions, deaths and positive tests. It takes two to three weeks for changes in the R rate to be shown in this data, due to the time gap between infection and the need for treatment.”

Third, key descriptive information has been often conveyed in the media via *text*, as in figures communicated as printed text or spoken language (e.g., interviews with experts), apart from numbers embedded in text. A rich example of this observation, which also illustrates area C in Table 2, is an *ABC* (Australia) media item “Working from home because of coronavirus? The ATO’s new tax ‘shortcut’ may leave you paying more” (^Item#3^Khadem, 2020), which describes and critiques the Australian Tax Office’s response to people working from home due to pandemic lockdowns. This item embeds financial figures, percentages, monthly rates, and both small and very large numbers in a predominantly text-based report.

### Category 2: Models, predictions, causality, and risk

This category of StaMPs in media items is concerned with discussion about mathematical and statistical models in terms of making predictions about the progress of the pandemic, the causal relationships between relevant variables, and the estimated chances and risks associated with the results of different courses of action (e.g., policies regarding adopting mask wearing or lockdown measures) or inaction.

A model is a simplification and representation of reality that incorporates essential features of a real-world situation and the relationships between them (Geiger et al., 2022). Statistical models are used for predicting the progression of many phenomena including the progress of epidemiological events. Models are complex tools, and their outputs must be interpreted with caution because of their reliance on assumptions and the quality and quantity of available data (Steyerberg, 2019).

Many model-based predictions and targets were reported in the sampled media items, for example, the projected progression of the pandemic over time in terms of the number of infections, people in intensive care, or deaths. Modelling has also been used to predict the potential effect of various interventions (e.g., social distancing) on the progression of the pandemic to avoid available medical resources being overwhelmed (i.e., how to “flatten the curve”) or to project when herd immunity can be achieved.

Due to their predictive power, models have been used by governments, taking account of the advice of experts, as the basis for formulating policies aimed at controlling the pandemic and alleviating its social and economic impacts. The public visibility of discussions about models and modelling has been evident across all news outlets in our sample, for example, in media items reporting about press briefings and statements by heads of state (presidents, prime ministers), key public figures and various experts. These aspects of COVID-19-related reporting are illustrated in an *ABC* report presented in Table 4 (^Item#6^Hayne, 2020).

**Table 4:** Models, predictions, and vagueness in a media article

Coronavirus “nowcasting” modelling shows Australian case numbers continue to fall
…as health authorities continue to refine modelling of Australia’s coronavirus curve, the margin of error in their findings is getting wider — but that’s actually a good thing. Chief Medical Officer Brendan Murphy presented the latest coronavirus modelling on Friday afternoon, showing that Australia continues to stamp down the number of new infections being recorded each day
Currently, there have been 6,675 confirmed cases of COVID-19 in Australia, of which more than 5,000 have recovered. There have been 78 deaths
**Margins of error are getting wider**
The Government’s “nowcasting” of the coronavirus situation in Australia aims to take stock of the coronavirus situation using the latest numbers. But that process gets less accurate if less data is fed into it. As case numbers continue to fall, the data being put into modelling is shrinking, making forecasts less precise. That being said, predictive modelling looking at the coming two weeks does suggest a further decline in numbers…

**Fig. 1:**
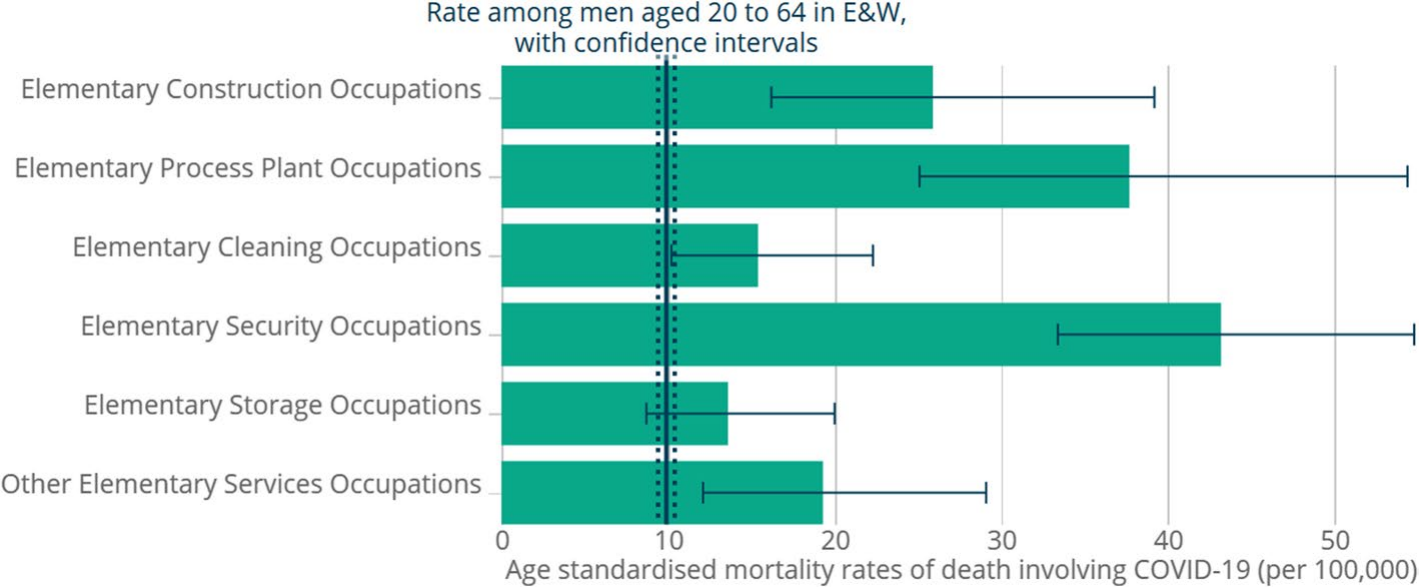
Risk-related graph from Office of National Statistics (UK) open- source report, *The Sun* (^Item#7^Burrows, 2020)

Our analysis identified four separate nuances on the ways media items discuss the results or uses of modeling:

- First, discussions of predictions were often intertwined with commentary on cause-and-effect relationships or long-range consequences of the pandemic.
- Second, discussions of modelling were also intertwined with explicit references to the *vagueness or error* inherent in predictions based on modelling.
- Third, there were numerous instances (Table 5: examples 1, 4, 14] of different predictions for the same phenomena (e.g., expected deaths), presumably all based on credible models. Such media items sometimes exposed the difference between models in terms of underlying assumptions or variables.
- Fourth, the results of modelling often used language related to *risk* (Glik, 2007), including situations associated with the likelihood of infection or death (see Fig. 1), or the chance of long-term health effects, and associated consequences.

**Table 5:** Headlines from four news outlets, related to data quality, variability, comparability, stability, credibility, doubts and vagueness, and strength of evidence

#	*Source*	Date	Headline (title) of article
1	*CNN* article (^Item#8^LeBlanc, 2020)	Apr 8, 2020	US coronavirus predictions are shifting. Here’s why
2	*CNN article* (^Item#9^Liptak and Acosta, 2020)	May 13, 2020	Trump privately questions whether coronavirus deaths are being overcounted as Fauci projects the opposite
3	*CNN* article (^Item#10^Ilyushina and Hodge, 2020)	May 14, 2020	Why Moscow didn’t count 60% of suspected Covid-19 deaths
4	*The Sun* article (^Item#11^Tahir, 2020)	Apr 28, 2020	DEATHS MOUNT: Sweden suffers worst week for deaths this CENTURY despite claiming it had passed coronavirus peak without lockdown
5	*The Sun* article (^Item#12^Rogers, 2020)	May 3, 2020	GRIM TOLL: UK coronavirus death toll could be 45,000…nearly DOUBLE the official figure, statistics expert says
6	*The Sun* article (^Item#13^Vonow, 2020b)	May 8, 2020	RISK FACTOR: Black Brits FOUR TIMES as likely to die from coronavirus as white people, shocking new figures reveal
7	*The Sun* article (^Item#14^Carter, 2020)	May 18, 2020	TOO SOON: Government will NOT vary coronavirus lockdown rules by region despite wide-ranging infection rates across parts of UK
8	YNET* article (^Item#15^Gal, 2020)	May 22, 2020	Contrary to prior warnings, the CDC claims: Coronavirus does not transmit easily via surfaces
9	YNET* article (^Item#16^Alon, 2020)	May 31, 2020	Numbers of migrants infected with corona higher than reported: Afraid to get tested
10	YNET* article (^Item#17^Janco, 2020)	Jun 7, 2020	Report: Gaps between government Ministries in coronavirus deaths figures
11	*ABC* article (^Item#18^Willis, 2020)	April 3, 2020	Face masks debate: WHO consider evidence for widespread face mask use, as US set to change its advice
12	*ABC* article (^Item#19^RMIT ABC Fact Check, 2020)	April 26, 2020	Fact Check: There’s still no evidence 5G is spreading coronavirus, and other COVID-19 misinformation
13	*ABC* TV/video report(^Item#20^Harnik, 2020)	May 4, 2020	Mike Pompeo says ‘significant’ evidence new coronavirus emerged from Chinese lab
14	*ABC* radio broadcast/podcast (+ online transcript) (^Item#21^Swan, 2020b)	May 11, 2020	Modelling the mental health impacts of COVID-19
15	*ABC* radio broadcast/podcast (+ online transcript)(^Item#22^Swan, 2020a)	June 1, 2020	Coronavirus testing errors, streamlining outbreak data, chronic fatigue, and who we trust

^*^Note: YNET articles translated from Hebrew

These nuances are all related, which explains why models, predictions, causality and risk are intertwined into a single category of StaMPs. The nuances outlined here also seem essential for understanding proposed policy interventions (Ridgway, 2021).

### Category 3: Visual representations and displays

Visual representations of data and statistics related to the pandemic and its progression were found in media items from all four news outlets, in line with the relevant research literature (e.g., Friel et al., 2001; Spiegelhalter et al., 2011). Many media items, but not all, included static visual representations of data including line graphs (linear and logarithmic displays), pictographs, column graphs, box plots and pie graphs, and tables. A few also contained dynamic displays such as interactive maps and visualizations that enabled users to select topics or parameters and view the progression of different aspects of the pandemic over time or across geographical locations.

Surprisingly, visual representations and displays seldom took central stage within the media items we analyzed. Instead, it is text, or numerical figures embedded in passages of text, that were primarily used to convey statistical and mathematical information or trends about the pandemic. Such texts included descriptive information from the four areas included in category 1 or the results of other analyses. This means that the trends or key issues that readers could glean from any graph or display were typically explained in the text, at least in a general way, making it unnecessary for readers to examine the visual display itself in detail. Yet, *The Sun* (UK) and *YNET* (Israel) often employed a strategy where 2–4 visual displays (e.g., a simple line graph, bar chart or heat map showing infection or death counts or R-rates) are used in an undifferentiated way, that is, the same daily displays are inserted into many pandemic-related media items, regardless of their actual topic.

That said, some media items employed more sophisticated representations, sometimes produced by the media outlet itself, but often imported from an official statistics source or from a statistics integrator (see Category 8 in Section 4.8). An example can be seen in Fig. 1, taken from *The Sun* item “WORKERS AT RISK: Taxi drivers, shop staff and security guards most likely to die from coronavirus but medics NO more at risk than public” (^Item#7^Burrows, 2020). Here, elevated risks of infection and death for particular groups were discussed, using a graph from a UK Office of National Statistics report published earlier that day.

The display in Fig. 1 shows a seemingly simple bar graph but has additional textual and visual information referring to “age-standardized mortality rates” (a complex indicator, not explained in the text), confidence intervals bars and a vertical line showing the average for the age group in the general population against which the bars should be compared. Thus, the display demands attention to and comprehension of multiple statistical elements and ideas. The authors of *The Sun* article attempt to help readers gain some insight into the meaning of the graphs via a simple title under the graph: “Security guards were most likely to die from Covid-19, ONS figures reveal.”

In summary, most media items appeared to use displays that add visual interest but carry relatively little unique informational value, since the “take home” messages are usually provided in the text itself, confirming arguments from journalism literature that numbers often serve as rhetorical devices (Roeh & Feldman, 1984). However, as illustrated above, some media items have used complex representations, showing comparative or multivariate information that support the need for sophisticated levels of statistical literacy *and* official statistics literacy (Gal & Ograjenšek, 2017). This confirms conclusions by Kwon et al. (2021), who analyzed graphs in news stories related to COVID-19 and argued that such graphs are often outside the scope of the mathematics taught in schools. This argument is consistent with earlier claims about the diverse cognitive demands of statistical products from official sources (Gal & Murray, 2011).

### Category 4: Data quality and strength of evidence

Media items in our sample also referred to two related themes—*data quality* and *strength of evidence*. These two themes are illustrated via Tables 5 and 6 (Note: The given examples refer to full media items, not just to the headlines listed).

**Table 6:** Excerpt from CNN article: “Scotland recommends face coverings as cracks emerge in UK-wide approach to coronavirus” (^Item#23^Rahim, 2020)

Scotland’s First Minister said… “The evidence on the use of face coverings is limited, but there may be some benefit in wearing a facial covering when you leave the house and enter enclosed spaces.”…
[In England] the Scientific Advisory Group for Emergencies (SAGE) have submitted evidence to ministers, and the government would announce a decision as soon as it was made, the Prime Minister's official spokesperson said,…
Deputy Chief Scientific Adviser Angela McLean said SAGE had concluded there was “weak evidence of a small effect” in which face masks could prevent an infected person passing Coronavirus on to someone else

The theme of *data quality* emerged from media articles (Table 5, examples 2, 3, 5, 9, 10, 15) that discuss different types of data collection and measurement errors, technical problems, or other methodological issues that affect the credibility or validity of the data that underpin the StaMPs described in categories 1–3. Such issues are then positioned as a reason to question the soundness or validity of the *interpretations* or the *conclusions* drawn from StaMPs. Examples that require this type of scrutiny include changed definitions of what counts as “death from coronavirus,” faulty infection testing procedures, and over/under-counting of infections or deaths in some subgroups that lead to erroneous conclusions regarding the pandemic’s rate of progression.

Our observations about references to data quality in media items both corroborate and extend existing arguments that readers should be able to interrogate the quality of data presented, for example, in surveys (Gal, 2002). We further note that while some of the issues listed above are typically covered in introductory statistics textbooks, for instance, error due to sampling or survey non-response, some are addressed only in advanced sources discussing official statistics (Radermacher, 2020).

Other media items focus on the related but separate theme of *strength of evidence*. Within several media items, the need for evidence is discussed as a stand-alone construct, as illustrated in the excerpt in Table 6, which outlines the (lack of) evidence for the contentious requirement to wear face masks. There were also media items that referred to the quantity of evidence or to its strength or credibility (i.e., being inconsistent, inconclusive, ambiguous). Such items (Table 5: examples 4, 11, 13) typically argue that inconclusive evidence cannot support policy changes—but without providing specifics of the nature of that evidence or its actual limitations.

While the two themes of data quality and strength of evidence are separate, they are related. Together they illustrate that readers should be able to understand the tentative nature of pandemic-related statistics and predictions, and that raw data, analyses and results are contestable or subject to revision. This perspective challenges the traditional view of scientific findings or statistics being truth-laden (Brandao, 2017).

### Category 5: Demographics and comparative perspective

Understanding the progression of the pandemic requires both a comparative perspective (Best, 2020) and a global view of events. These are important 21st century skills (Griffin & Care, 2015; Maass et al., 2019). StaMPs included in this category require that data be compared across age groups, neighborhoods, cities, states/provinces or countries (Table 5: examples 6, 7), in addition to gross national summaries. Obtaining a comparative insight into the severity of the pandemic, or the outcomes of different policy and health strategy responses, requires that readers understand both geographical information, maps, and associated underlying population demographics. In particular, the impact of population size, in both absolute and proportional terms, must be understood to compare the scope of the pandemic using the same basis for calculation, as when media items compare infection levels by reporting about “positive cases per 100,000 inhabitants” or similar ideas. While seemingly simple, such comparisons and ratios are nonetheless not covered in regular statistics curricula but only common in official statistics resources (Radermacher, 2020).

### Category 6: Heterogeneity and contextual factors

StaMPs included in this category were used to report quantitative information about the relative size or distribution of certain groups with specific characteristics, such as religious status, economic capabilities, or cultural or attitudinal aspects. Such StaMPs are grouped as a separate category because they form “softer” contextual correlates (Ancker, 2020) whose understanding is essential for credible interpretation of reported figures and statistics related to a pandemic, beyond those included in categories 1–5. For example, our sample included media items about elevated infection levels for some religious groups who did not follow social distancing regulations, or reports about resistance to testing or to masking regulations among migrants, “anti-vaxxers” and other communities (Table 5: examples 5, 6, 9). Media items also documented (Table 5: example 12) rumors and fake evidence circulating on social networks that resulted in a defiant attitude toward social distancing requirements in some (virtual) communities. In addition, some items discussed the willingness of specific social groups to accept potentially unreliable reports, or “fake news” (Table 5: example 2) that involved misleading statistics, affecting their compliance with regulations during the pandemic (see also Yoon et al., 2021).

Such contextual correlates involve information that has to be taken into account by citizens (and policy makers) when they interpret StaMPs included in categories 1–5. First, heterogeneity and contextual factors can affect data quality, for example, if some groups do not agree to be tested, estimates of infection levels for them are likely to be inaccurate. Second, these factors may also impact the accuracy of *predictions* related to the pandemic, for example, if some subgroups do not comply with public regulations in the ways assumed by the modelers, due to their beliefs, economic needs or other characteristics. Finally, such correlates may refer to statistics associated with equity or fairness issues related to *consequences* of the pandemic, as when StaMPs discuss higher mortality rates in minority groups (Table 5, example 6).

### Category 7: Literacy and language demands

This category captures the realization that the primary mode of communication associated with StaMPs in media items about the pandemic is *text*, either written text (e.g., news articles) or spoken text (e.g., speeches by public officials, interviews with experts). The pandemic has also introduced new language demands into the public arena because of the need to communicate a wide range of mathematical and statistical ideas, as illustrated by the excerpts in Table 7. Such and related examples illustrate that citizens must acquire and activate sophisticated text comprehension capabilities if they are to access and understand descriptive quantitative information and more advanced mathematical and statistical ideas within media items (e.g., regarding modelling; Krawitz et al., 2022). Furthermore, an awareness of language and literacy demands is key to comprehending news and professional jargon as well as to its (cautious) interpretation. Such demands are consistent with an established body of literature devoted to the role of language in everyday numeracy and statistical literacy (e.g., Bertotti, 1941; Gal, 2002). Yet, language-minority groups and those with limited educational backgrounds or literacy skills are thus more vulnerable in terms of access to pandemic-related information (Gal et al., 2020).

**Table 7:** Excerpts illustrating the role of language and text in mediating statistical and mathematical ideas to the public, and the demand for critical interpretation

1	Source: *ABC, radio interview with an epidemiologist (See Table **5**, Example 14):*
	“The COVID-19 pandemic has hugely increased our data literacy. Words and phrases like modelling, exponential growth, epidemic curves and reproductive values and false negative rates just trip off our tongues as we've had little else to do in isolation than watch the statistics day by day.”
2	Source: *The Sun,* ‘Coronavirus Explainer’ section. “CORONA RATING: What is the current R rate in my area? How to track it every day?” (^Item#24^Knox, 2020)
	The infamous “R” rate is currently guiding the Government’s coronavirus response, as each of the planned phases depend on how infectious Covid-19 is. The rate has emerged during several Downing Street briefings, with both officials and the Prime Minister
	**What is the R rate in your area?** Different parts of the UK have a different R infection rate—which is used to indicate the speed the virus is spreading. In the latest coronavirus lockdown review Sir Patrick Vallance, the chief scientific adviser, said the current R rate across the UK is between 0.7–0.9
	But he did caution that it may be very close to 1 in some places, and said it means that “54,000 new cases are occurring every week”. “That is not a low number, so it's worth remembering that we still have a significant burden of infection, we are still seeing new infections every day at quite a significant rate and the R is close to one,” he added

### Category 8: Multiple information sources used by the media

There were media items in our sample that utilized data-based statements and visual representations from a wide range of sources. These included established organizations that assemble multinational data based on shared reporting schemes, for example, national producers of official statistics (e.g., Office of National Statistics [ONS, UK], Centers for Disease Control and Prevention [CDC, USA]) and international agencies (e.g., World Health Organization [WHO]). Furthermore, institutions which we term “statistics integrators” have received worldwide recognition in the media during the COVID-19 pandemic. Such integrators include centers at academic institutions (e.g., *Coronavirus Resource Center* at John Hopkins University, USA, https://coronavirus.jhu.edu; *Our World in Data*, Oxford University, UK, https://ourworldindata.org/coronavirus), commercial organizations (e.g., *Worldometer,* www.worldometers.info), and academic research centers.

Interpreting StaMPs in media items thus requires a sophisticated understanding of the role of these different institutions and integrators, as well as attention to the trustworthiness of the information they generate. A pertinent example can be found in a *CNN* article (^Item#1^Mclean et al., 2020), which describes how Spain’s Prime Minister appeared on national television to report on the nation’s status in relation to testing for virus infections and cited infection rankings published by *Worldometer*, a source that *CNN* claims is not recognized.

### Category 9: Critical demands in StaMPs

There is an increasing emphasis in the literature on mathematics, statistics, and numeracy education regarding the need for citizens to develop critical capabilities. D’Ambrosio (1999) and Skovsmose and Nielsen (1996), for example, argue that critically evaluating the arguments and propositions of authority is vital for the development of an ethical, moral, and socially just society. This means that citizens, when engaging with media items, must have the capability (i.e., knowledge and disposition; Gal, 2002) to question and react to the propositions and arguments they encounter.

Indeed, the analysis showed that all the categories of StaMPs identified within pandemic-related media items could be the subject of critical scrutiny. For example, the comparability of figures is noted in category 1 (see Section 4.1 on item “Explanation about the R-Rate”, *The Sun*), the assumptions that underpin models or projections of future progression of the pandemic in category 2, and the use of potentially misleading graphs in category 3. A critical eye is also needed for judgements about data quality and strength of evidence in category 4, the validity of group comparisons across different demographic groupings or contextual factors in categories 5–6, and the other aspects of StaMPs as noted in categories 6–8. Thus, while *critical demands* is a distinct construct within the typology, it is intertwined through each of the other eight categories of StaMPs.

We have also identified, however, a separate phenomenon that has not, to our knowledge been reported before, termed here “embedded criticality,” in which the news item text itself directs readers to statistical and mathematical issues that require critical scrutiny. In order to understand the concerns being raised in an item by a reporter, readers must be capable of reading and comprehending the underlying mathematical and statistical arguments being discussed. For example, some media items refer explicitly to problems with data quality or strength of evidence, contrast different predictions about the same phenomena, highlight disagreements between experts, discuss “fake news” being circulated, or present outcomes of “fact checking”—all of which suggest that reported claims or arguments may contain inaccuracies or errors. Texts with embedded criticality, in the way described here, as in several headlines in Table 5 and quotes in Tables 4, 6, 7, explicitly imply that citizens have reason to challenge mathematical or data-related statements and directly invite citizens to be critical.

Some media items in our sample go further by questioning the quantitative logic underlying *policy decisions* and the evidence made available to justify their *consequences*. For example, Table 5, examples 6, 1, illustrates how the media challenges the logic underlying the imposition of restrictions or “lockdowns” and then rationalizing the easing of social distancing or related constraints. Although the need to consider the consequences of decisions made on the basis of mathematical and statistical arguments has been raised previously (e.g., D’Ambrosio & D’Ambrosio, 2013; Madison & Steen, 2003; ProCivicStat Partners, 2018; Skovsmose & Nielsen, 1996), it is not usually included in the literature on statistical literacy or adult numeracy (e.g., Weiland, 2017).

### Overview and the inter-relatedness of categories of StaMPs

**Fig. 2:**
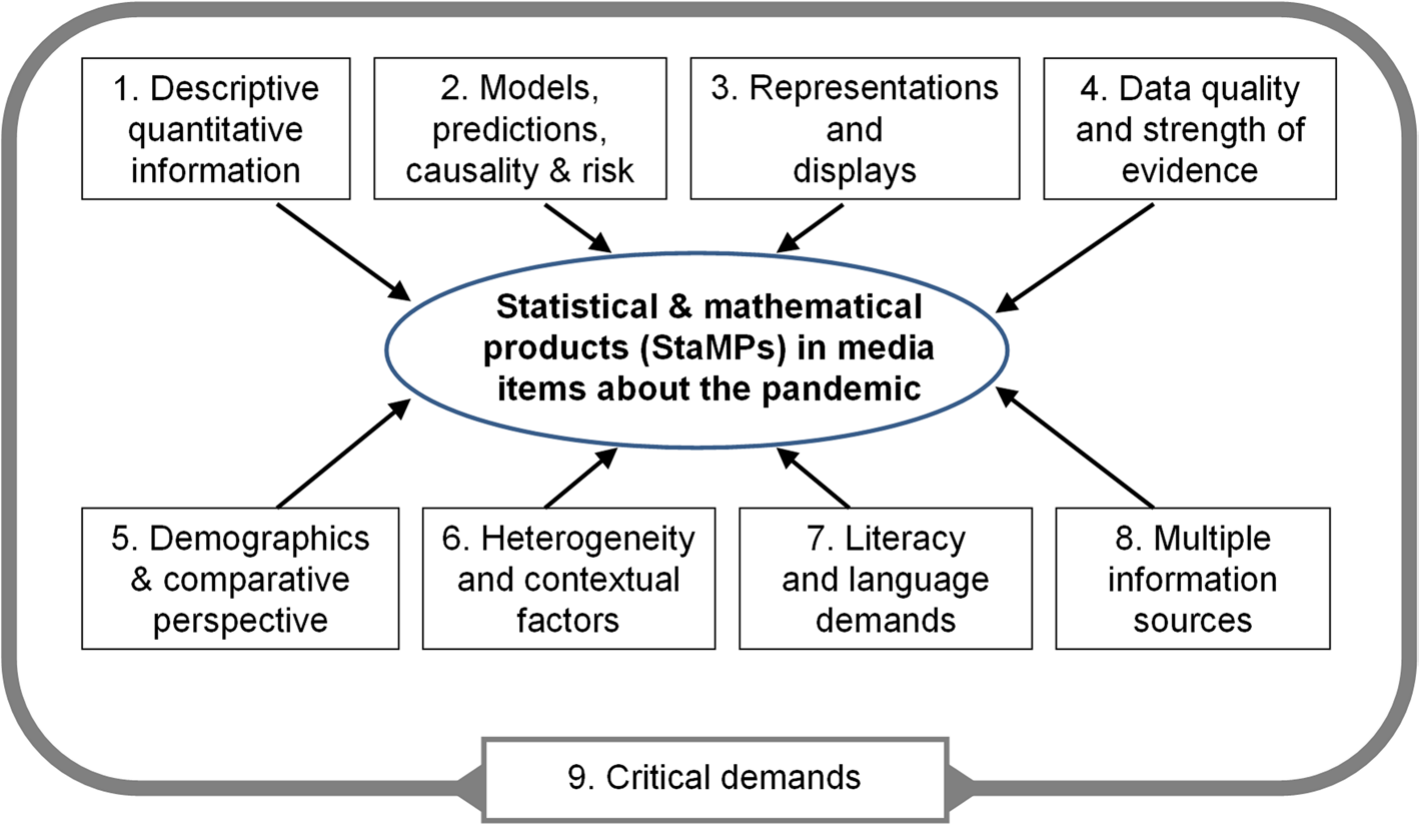
Nine categories of StaMPs in pandemic-related media items

In closing the findings section, we present Fig. 2 which provides an overview of the nine categories of StaMPs in media items. We see categories 1–8 as identifiably discrete but often interrelated, since a single media item may encompass content from multiple elements. Furthermore, category 9, critical demands of StaMPs, can be relevant to or applied to any of the other eight categories when interrogating a media item.

Below we present four examples which emerged from our sample that illustrate the inter-related nature of the nine categories of StaMPs:

- Descriptive quantitative information about the pandemic's progression (category 1, see Table 3) also involves routinely reported figures regarding statistical indicators such as the R rate or positivity rate, which in fact are aggregation models (category 2). Such figures are often reported via various representations (category 3), as when “heat maps” compare infection levels in different cities.
- StaMPs are used to present comparisons and predictions while referring to the results of modelling (category 2) but are sometimes intertwined with ideas related to strength of evidence or data quality (category 4) as when the results of modelling are critiqued (category 9).
- Predictions (category 2), and statements about risk (e.g., of contracting the virus) are often reported via text only (category 7; as in an expert interview), and less often through graphical representations (category 3).
- StaMPs demand knowledge about demographics (category 5) or other contextual factors (category 6), for example, relative size of certain social groups and their geographic dispersion, to be able to think critically (category 9) about the link between available data, data quality (category 4) and the soundness of resulting policy decisions.

## Discussion

In this study, we have aimed to contribute to knowledge related to the characteristics and demands of statistical and mathematical products (StaMPs) across diverse types of media items. Addressing the current knowledge gap in this area can enhance the evidence base by identifying the capabilities or essential competencies that underpin citizens’ capacity to evaluate the meaning, credibility, significance and relevance of information they encounter in the news media (cf. Glik, 2007).

This section examines four separate contributions of the findings of the present study to the research literature on mathematics and statistics education as well as adult numeracy, and discusses implications for research and practice.

### Contribution #1: A data-based typology of demands of statistical and mathematical products in the media

This study mapped categories of StaMPs within a large sample of media items related to the COVID-19 pandemic sampled from four diverse media outlets situated in different countries. This approach has extended the work of studies with a similar focus (Aguilar & Castaneda, 2021; Jablonka & Bergsten, 2021; Kwon et al., 2021), both in terms of sample size, the diversity of media items, and methodology.

Our analysis identified and created a new typology of nine categories of StaMPs that citizens encounter in pandemic-related media, which corroborates existing conceptual frameworks, while also extending current theory. For example, Gal’s (2002) model of statistical literacy posits the need for five interrelated knowledge bases, involving literacy skills, mathematical skills, statistical skills, contextual (world) knowledge and critical skills. The nine categories of StaMPs, summarized in Fig. 2, are inclusive of all these topics, at some level, but provide important *additional* elaboration beyond Gal’s (2002) model or frameworks related to adult numeracy (e.g., Tout et al., 2021), in particular regarding: models, predictions, causality, and risk (category 2); data quality and strength of evidence (category 4); understanding demographics and a comparative perspective (category 5); knowledge of some aspects of official statistics (categories 1 and 2); and multiple information sources used by the media (category 8). The ninth category, critical demands of StaMPs, is also not a stand-alone topic but intertwined with the other eight.

The additional or elaborated categories identified in this study provide a more sophisticated and multi-faceted perspective than in previous frameworks. Thus, the characterization of nine categories of StaMPs contributes new knowledge from which the mathematical and statistical capabilities needed to critically interpret and understand media items can be developed. Promoting such capabilities is essential because of the increasing likelihood of another pandemic, as a consequence of global commerce, travel, migration and urbanization (Gössling et al., 2020). However, such capabilities can be of relevance to citizen’s understanding of other societal crises (Topper & Lagadec, 2013) and civic topics as well.

### Contribution #2: The need for blended knowledge

The interrelatedness of the nine categories of StaMPs (Section 4.10) means that pandemic-related media items demand that citizens be capable of *integrating* different types of mathematical and statistical knowledge (e.g., number sense and statistics), representations of comparative or multivariate information and indicators commonly used in official statistics (Gal & Ograjenšek, 2017; Radermacher, 2020). We note that within school mathematics curricula, statistics are often taught as a separate topic from mathematics, yet in the media items, these knowledge bases are often intertwined or blended. The need to be competent with blended knowledge was particularly apparent in items that included discussion about technically complex issues, such as the use of modeling to justify claims related to prediction, causality and risk. Furthermore, to understand and interpret StaMPs in media items, readers or viewers need adequate literacy skills (category 7) to critically comprehend text with blended mathematical and statistical content.

Media items about the pandemic were often related to topics beyond health including the economy, unemployment, education, environment, and social equality. Hence, blended knowledge also covers contextual factors or correlates (categories 5 and 6), which require readers and viewers to engage different categories of StaMPs within a single media item, yet the types of capabilities required in this circumstance are typically taught in separate STEM disciplines. This finding is consistent with the conclusions of Kwon et al. (2021) that graphs related to COVID-19 that appear in items from South Korea were often outside the scope of the mathematics taught in schools. The need for blended knowledge, as identified in this study, provides empirical support for the conceptual framework proposed by ProCivicStat Partners (2018) regarding the capabilities needed for engaging with civic statistics topics.

### Contribution #3: Going beyond probability to vagueness and risk

Mathematics has often been portrayed as a way of representing reality that communicates fact and truth in a clean-cut, objective manner. For the media, this perspective of mathematics provides credibility for propositions and arguments in a seemingly unbiased fashion (Himmelstein, 2014). Similarly, in typical mathematics and statistics curricula, students learn that levels of probability associated with the occurrence of events are always quantifiable, that is, the chance of uncertain events can be accurately calculated and represented numerically (Pratt & Kazak, 2018). The uses of StaMPs in media items about the pandemic, however, often involve notions of *vagueness* and *risk*. For example, some media items refer to “insufficient evidence” or “high (or low) risk.” Similarly, media items about policy decisions (e.g., requirements to wear masks) are often reported as if they rely on quantitative information, yet, the relevant numerical results or specific figures are *not* necessarily communicated. This leaves a sense of ambiguity or vagueness about the meaning of quantitative or probabilistic phrases used in such media items.

These observations are consistent with that of Spiegelhalter et al. (2011), who noted the gap between the manifestations of *probability* in curricula and the expression of *uncertainty* in communications to the public. We have identified this gap in media items about the pandemic—at a time when the public’s understanding of risk and uncertainty has never been more crucial. This gap has implications for practice as it indicates that further thinking is required about the promotion of probability literacy (Gal, 2005) among school-age learners and adults (Lewis, 2021). We argue that such thinking could focus on understanding *levels of certainty* (or vagueness) and related influencing factors, such as quality of the data, sources of evidence, assumptions underlying model-based predictions and what critical “worry questions” (Gal, 2002, 2005) to raise when seeking clarification.

### Contribution #4: Expanding notions of criticality about evidence

Research related to critical thinking has a significant history within mathematics education, especially in relation to attempts to identify the capabilities or basic skills required by citizens to participate in society in an informed and responsible way (e.g., Madison & Steen, 2003). Critical thinking (Ennis, 2011) is seen as a key enabler of citizens’ capacity to form effective judgments and make prudent decisions (e.g., D’Ambrosio & D’Ambrosio, 2013; Ernest, 2002; Gal et al., 2020; Geiger et al., 2020; Skovsmose & Nielsen, 1996) and is a key element within models of numeracy and statistical literacy (e.g., Gal, 2002; National Academies of Sciences, Engineering, and Medicine, 2016; Tout et al., 2021). Recent analyses of the pandemic-related media (e.g., Borba, 2021; Jablonka & Bergsten, 2021; Stephan et al., 2021) have heightened the focus on criticality through attention to the role of mathematics and statistics in issues such as the social construction of reality, the shaping of public discourse about restrictions of citizens’ rights, or validating positions of power and political influence.

Our analysis of the critical demands associated with StaMPs within media items contributes to new knowledge in several ways:

1. The sophisticated manner in which mathematics and statistics are now used in the media means that citizens require not only knowledge of the limitations of the methods used to collect and analyze data, but also a deeper understanding of both how evidence underpins policy decisions and the consequences of their enactment (e.g., from the local community to the global economy).
2. Citizens must be able to comprehend and evaluate what we termed embedded criticality, that is, the critical positions or criticisms embedded in the media items themselves. This raises the need to rethink and enhance reading comprehension and critical reading skills related to mathematical and statistical arguments (Krawitz et al., 2021).
3. The mathematical and statistical demands of pandemic-related media items point to the need for capabilities associated with the notion of evidence literacy. Evidence literacy has been discussed in fields such as health education (e.g., Martin et al., 2017) and recently in science literacy (Duncan et al., 2018) but not previously considered a dimension of mathematical or statistical literacy nor included in mathematics or statistics curriculum frameworks (Geiger, 2019).
4. We have demonstrated that critical demands can be linked to all categories of StaMPs within our typology. This implies that the range of “worry questions” that citizens should be able to pose about media items should be extended, compared to prior frameworks (Gal, 2002, 2005; Weiland, 2017).

Identifying a need to develop the capacity to scrutinize the quality of “evidence,” and how it is used in the media, represents a contribution to new knowledge that extends previous understanding of what is required for informed, responsible and critical citizenship. This capacity is vital for evaluating the opinions offered by experts and knowing what questions to ask in order to form personal judgements and make decisions (e.g., Fischer, 2000; Kollosche, 2021; ProCivicStat Partners, 2018). Furthermore, the capacity to evaluate the quality of evidence is also key to disrupting the way a crisis might be formatted in the media, as described by Skovsmose (2021), and to developing a critical understanding of the rationales underpinning policy decisions and their justifications (Berger et al., 2021).

### Research and educational implications

Our findings about the broad and rich demands of StaMPs in the pandemic media have significant implications for teaching and learning in relation to developing citizens’ capacity to engage with numerate environments and practices (European Commission, 2018; Ferrara et al., 2017). These implications are relevant to all contexts of formal and informal education across a diverse range of information needs (Gal & Murray, 2011).

The outcomes of this study also have implications for future research and educational agendas, such as the following:

1. Refining our typology of categories of StaMPs that citizens encounter in the media, including their demands and characteristics. This requires the extension of the current categories to accommodate the demands of media items on other significant civic topics, such as global warming or equity, and using additional and more diverse sources of media outlets. Furthermore, it is important for future studies to go beyond analysis of text (print or digital) and examine the nature of StaMPs communicated via gestures (see Aguilar & Castaneda, 2021) and oral (spoken) modes of expression, such as by experts or presenters on TV news, radio programs, or podcasts.
2. Promoting educational programs aimed at developing the capability of citizens to use blended knowledge and critically evaluate both the meaning and strength of evidence that are used to support the claims and arguments of experts and other commentators in the media. An example is developing the ability to evaluate whether evidence from mathematical and statistical modelling is used in a credible way in the media, when predicting the progression of a crisis.
3. Generating educational programs that go beyond simple and abstract notions of probability, and include comprehension of levels of vagueness, uncertainty and risk as they are communicated in the media.
4. Examining citizens’ actual practices and behaviors (Gal et al., 2020; Geiger et al., 2020) when engaging and evaluating the different categories of statistical and mathematical products, i.e., StaMPs, in media items that report on key civic topics (e.g., a pandemic, global warming) and on related policy decisions.

The above implications illustrate key foci for research and education that are of relevance to both current circumstances and future crises (Barchas-Lichtenstein et al., 2021; Skovsmose, 2021; Topper & Lagadec, 2013). Ongoing work on the topics outlined in this article is essential to enable mathematics and statistics education to extend beyond existing theoretical models and serve the expanding needs of diverse societies around the globe.
